# Length of positive surgical margins after radical prostatectomy: Does size matter? – A systematic review and meta-analysis

**DOI:** 10.1038/s41391-023-00654-6

**Published:** 2023-03-01

**Authors:** Athul John, Alicia Lim, Rick Catterwell, Luke Selth, Michael O’Callaghan

**Affiliations:** 1 Adelaide Medical School, The University of Adelaide Faculty of Health and Medical, Sciences, Adelaide, South Australia Australia; 2 Urology, Central Adelaide Local Health Network, Adelaide, South Australia Australia; 3 Flinders Health and Medical Research Institute (FHMRI) and Freemasons Centre for Male Health and Wellbeing, College of Medicine and Public Health, Flinders University, Adelaide, South Australia Australia; 4 Urology, Southern Adelaide Local Health Network, South Australia Prostate Cancer Clinical Outcomes Collaborative, Adelaide, South Australia Australia

**Keywords:** Prostate cancer, Cancer epidemiology

## Abstract

**Objectives:**

The prognostic capacity of positive surgical margins (PSM) for biochemical recurrence (BCR) is unclear, with inconsistent findings across published studies. We aimed to systematically review and perform a meta-analysis exploring the impact of Positive surgical margin length on biochemical recurrence in men after radical prostatectomy.

**Methods:**

A search was conducted using the MEDLINE, Scopus, Embase and Cochrane databases according to the Preferred Reporting Items for Systematic Reviews and Meta-analysis (PRISMA) guidelines. The quality of the studies was assessed using the Newcastle-Ottawa scale, and the protocol was registered in advance (PROSPERO: CRD42020195908). This meta-analysis included 16 studies with BCR as the primary outcome measure.

**Results:**

Studies used various dichotomised thresholds for PSM length. A subgroup meta-analysis was performed using the reported multivariable hazard ratio (Continuous, 3, and 1 mm PSM length). PSM length (continuous) was independently associated with an increased risk of BCR (7 studies, HR 1.04 (CI 1.02–1.05), I^2^ = 8% *p* < 0.05). PSM length greater than 3 mm conferred a higher risk of BCR compared to less than 3 mm (4 studies, HR 1.99 (1.54–2.58) I^2^ = 0%, *p* < 0.05). There was also an increased risk of BCR associated with PSM length of less than 1 mm compared to negative surgical margins (3 studies, HR 1.46 (1.05–2.04), I^2^ = 0%, *P* = 0.02).

**Conclusion:**

PSM length is independently prognostic for BCR after radical prostatectomy. Further long-term studies are needed to estimate the impact on systemic progression.

## Introduction

Positive surgical margins (PSM) traditionally represent an adverse surgical outcome. In men who have undergone radical prostatectomy, it occurs in 11–37% of cases [1, 2]. It is associated with a worse prognosis and a higher risk of secondary treatment compared with patients who have a negative surgical margin (NSM) [3, 4]. However, not all men with PSM experience these equivalent outcomes. Only 27–44% of men with PSM develop a biochemical recurrence (BCR), 6.8–24.3% develop systemic progression and 0.8–3.7% experience prostate-cancer-related mortality over a 7–13 year follow-up period [5–8]. Thus, better risk stratification is required for those with PSM to help predict those who will experience BCR and initiate secondary treatment appropriately.

Recently, there have been multiple studies investigating the margin extension or length of PSM and its impact on BCR. There are inconsistencies in recommendations based on studies investigating the length of PSM with various margin thresholds used to determine higher and lower risk groups (1 mm, 3 mm). Hence, we aimed to systematically review and perform a meta-analysis answering the clinical question: Does the PSM length (intervention and comparator) influence BCR (Outcomes) in men with PSM after radical prostatectomy (Population)?

## Methods

### Search strategy

A systematic search was conducted using the MEDLINE, Scopus, Embase and Cochrane databases. The review included studies published up to 31st March 2021 [9]. A further literature search was performed by examining reference lists of included studies identified from the search. The protocol was registered at the international prospective register of the systematic reviews database (PROSPERO: CRD42020195908). Search terms were identified and adjusted to match the requirements of each database with the assistance of a librarian.

### Inclusion criteria

Studies exploring the association margin length of PSM after radical prostatectomy in men with prostate cancer in predicting BCR or oncological outcomes were included (Table 1). The review followed the Preferred Reporting Items for Systematic Review and Meta-analysis Methods (PRISMA) protocol [10]. The search results were independently reviewed by two authors (AJ and AL), initially based on title and abstract screening, followed by a full-text review. Input from a third author (MO’C) was used to resolve disagreements between authors. Data extraction and risk of bias were conducted by two independent authors (AJ and AL).

**Table 1:** Summary of included studies for the review.

Study	Analysis Group, Cohort type	PSM size/Overall	Follow up - Median (Years)	Type of Procedure	Gleason score included	Stage included	Adjuvant	BCR Definition
Cao et al. [16]	C, S	336/3005	3.4 (Mean)	NR	6/7/8/9	pT2/pT3a/pT3b	Excluded	> 0.2 ng/ml
Holleman et al. 2019	C, S	284/835	4.5	NR	GG1/GG2/GG3/GG4/GG5	pT2/pT3a/pT3b/pT4	Excluded	2 readings > 0.2 ng/ml
Hsu et al. [18]	C, S	117/789	5.3(Mean)	Open	GG1/GG2/GG3/>GG4	pT2/T3a/T3b/T4	Excluded	> 0.2 ng/ml
Huang et al. [19]	C, S	238/1048	1.5	NR	7	pT2/pT3	Excluded	2 readings > 0.2 ng/ml
Kates et al. [20]	C, S	401/4082	1.8 (+/− 1)	NR	NR	pT1/pT2/pT3	30% Rtx for PSM	> 0.2 ng/ml
Kim et al. [21]	C, S	146/817	3.1(1.6–5.2)	Open/Lap/Robotic - 11/129/25	6/7/8/9	pT1/pT2/pT3	NR	2 readings > 0.2 ng/ml
Savdie et al. 2011	C, S	285/940	6.8 (0.42–12.2)	NR	6/7/> 8	pT2/pT3/pT4	27.4% of PSM cohort – Rtx or ADT	2 readings > 0.2 ng/ml
Dev et al. [15]	3 mm, M	486/4000	6.2	Robot	GG1/GG2/GG3/>GG4	pT2/T3a/>T3b	Excluded	2 readings > 0.2 ng/ml
Ploussard et al. [22]	3 mm, S	402/1504	2.75(1.6–3.7 IQR)	Lap	6/7/8–10	pT2/T3a/T3b-4	Excluded	2 readings > 0.2 ng/ml
Preisser et al. [14]	3 mm, M	579/8770	6	Open/Robot	GG1/GG2/GG3/GG4/GG5	NR	Excluded	2 readings > 0.2 ng/ml
Sevoll et al. 2014	3 mm, S	163/303	5.7(0.7–26)	Open	GG1/GG2/GG3/>GG4	pT2/pT3a/>pT3b	Excluded	2 readings > 0.2 ng/ml
Lee et al. [25]	NSM v < 3 mm	473/1733 (Asian population)	4.4	Open	GG1/GG2/GG3/>GG4	<T2b/T2c/T3a/T3b	Excluded	2 readings > 0.2 ng/ml
Maxeiner et al. [27]	NSM v 3	325/2155	7.1	Lap	< 7/7/> 7	pT2/pT3a/pT3b/pT4 or Node positive	Excluded	2 readings > 0.2 ng/ml or persisting
Martini et al. 2019	NSM v > 3 mm, < 3 mm, > 1 mm, < 1 mm	285/1757	3	NR	Gleason Grade1–3/4–5	pT2/pT3a/pT3b/4	Excluded	2 readings > 0.2 ng/ml, Clinical recurrence – Imaging protocol up to clinician
Sammon et al. 2012	1 mm	162/794	4.5	Perineal RP	6/7/8/9	pT2/3	Excluded	2 readings > 0.2 ng/ml
Sooriakumaran et al. 2014	NSM v < 1	189/904	min 5 years	Robot only	6/3+4/4+3/> 8 76/69/25/18	pT2/pT3a/pT3b (9.4/23.2/60.5)	Excluded	1 reading > 0.2

*C* continuous variable, *CI* confidence interval, *S* single centre cohort study, *M* multicentre cohort study, *NR* Not reported, *GG* Gleason grade group, *PSM* positive surgical margin, *NSM* negative surgical margin, *RP* radical prostatectomy, *Rtx* radiotherapy, *PGG* primary Gleason grade, *IQR* interquartile range, *Lap* laparoscopic, *BCR* biochemical recurrence, *PSM 3* mm: positive surgical margin length greater than 3 mm, *PSM 1* mm: positive surgical margin length greater than 1 mm.

### Study eligibility

The review considered all published studies, including randomised controlled trials, observational cohort studies and case-controlled studies. The language of publication was restricted to English. Covidence (Covidence systematic review software, Veritas Health Innovation, Melbourne, Australia) and EndNote 20 (Clarivate Analytics, Philadephia, USA) were used to track studies included and excluded from the review.

### Statistical analysis

Multivariable Cox proportional hazard ratios for BCR were extracted after being adjusted for preoperative PSA, Gleason score and stage. Studies were subdivided based on the various dichotomised thresholds of PSM length that were used for analysis. Our meta-analysis was performed in the following subgroups: NSM vs <1 mm PSM;NSM vs >1 mm PSM; NSM vs <3 mm PSM; NSM vs >3 mm PSM; and >3 vs <3 mm PSM. The heterogeneity of the selected studies was calculated using the I^2^ score. A random-effects model was adopted for the meta-analysis, which was performed using Review Manager Software version 5.3 (The Nordic Cochrane Centre, the Cochrane Collaboration, Copenhagen, Denmark).

### Assessment of bias

Since no randomised controlled trials were included in our systematic review or meta-analysis, the Newcastle Ottawa Scale for non-randomised studies was used to evaluate the risk of bias [11]. The scale was scored by two authors. Publication bias was assessed using visual inspection of funnel plots where there were 10 or more studies present.

### Grey literature

The search strategy yielded several published conference abstracts that discuss the length of PSM as a prognostic clinicopathological feature. Those that progressed to publication were included for review. For the remainder, the limited information prevented adequate assessment of the quality and statistical methods used and hence they were excluded.

## Results

**Fig. 1:**
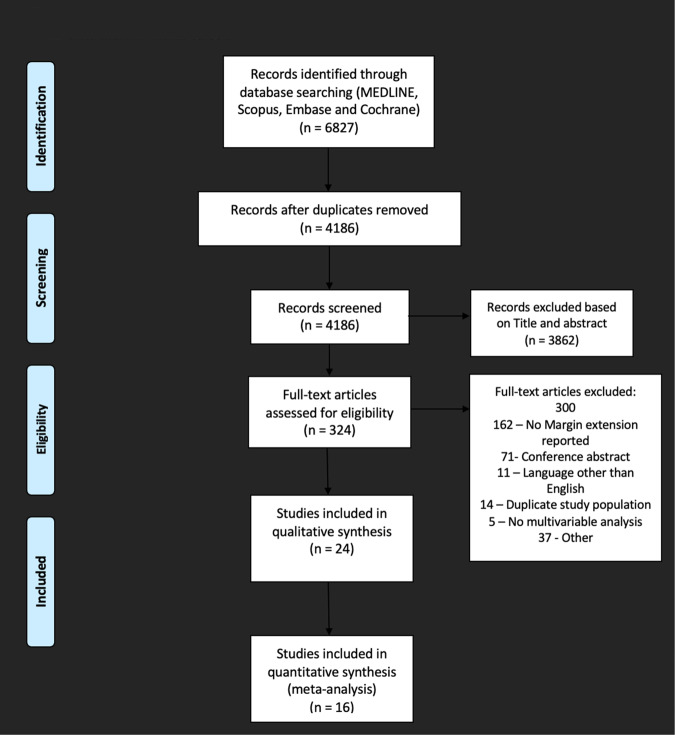
Preferred reporting items for systematic reviews and meta analyses (PRISMA) flow chart of chart of methodology.

The search strategy identified 6827 studies across multiple databases. After duplicates were removed and irrelevant studies excluded, 324 full-text articles were retrieved. Of the articles retrieved, 290 were excluded. Key reasons for exclusion included an absence of reporting on margin extension of PSM, abstract-only studies, duplicate study population and an absence of multivariable analysis. Of the 34 remaining studies, 10 did not report a Cox multivariable hazards ratio, and two were excluded due to a non-standard definition of BCR (PSA > 0.1 ng/ml) [12, 13]. Eight studies met the criteria but could not be included in a corresponding subgroup analysis due to non-standard PSM length thresholds. The remaining sixteen studies were included for meta-analysis (Fig. 1).

### Characteristics of the included studies

A summary of included studies for meta-analysis can be found in Table 1. All studies were retrospective cohort studies published between 2010 and 2020. Most of the studies were single-centre cohort studies, apart from two which were multicentre retrospective cohort studies [14, 15]. The median/mean follow-up period ranged from 1.5 to 6.8 years. PSM sample size ranged from 117 to 579 patients. Surgical techniques varied with three studies including robot-assisted laparoscopic prostatectomy only, three including open radical prostatectomy, two including laparoscopic radical prostatectomy and three studies including mixed techniques (RALP/RP/LP). Seven studies did not specify the technique. Studies used the standard EAU definition for BCR (serum prostate-specific antigen (PSA) measurements > 0.2 ng/ml) (Table 1).

There was significant variability in the reporting styles and dichotomised thresholds used to determine high and low-risk groups between studies: 7 studies reported the length of PSM as a continuous variable [3, 16–21]; 7 studies dichotomised the patients into 2 groups using a 3 mm PSM length threshold [14, 15, 22–27]; and 3 studies dichotomised the patients into 2 groups using a 1 mm PSM length threshold [24, 26, 28].

### Length of positive surgical margin and biochemical recurrence

**Fig. 2:**
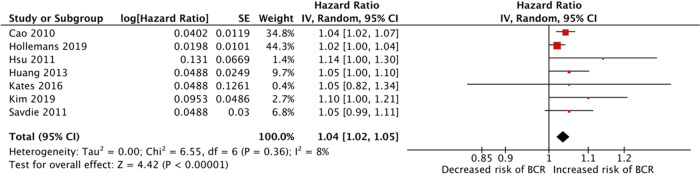
Forrest plot demonstrating biochemical recurrence risk associated with length of positive surgical margin (continuous variable). CI confidence interval.

**Fig. 3:**
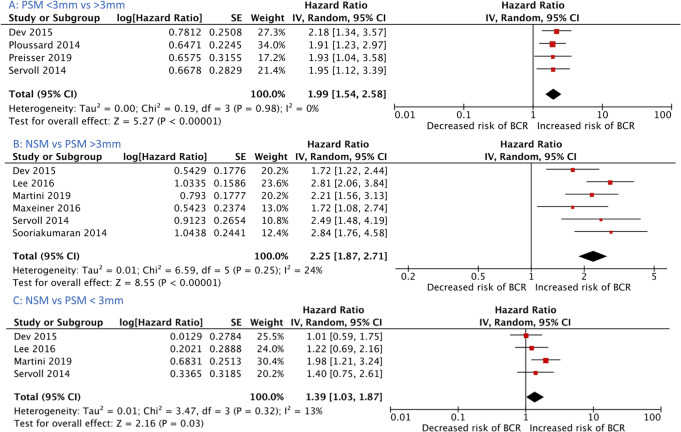
Biochemical recurrence risk for studies dichotomising margin length using a 3 mm threshold. **A** Compares BCR risk for studies comparing PSM length 3 mm. **B** Compares BCR risk for studies comparing NSM and PSM > 3 mm. **C** Compares BCR risk for studies comparing NSM and PSM. CI confidence interval, PSM < 3 mm positive surgical margin length less than 3 mm, PSM > 3 mm Positive surgical margin length greater than 3 mm, NSM negative surgical margin.

Increasing linear PSM length (continuous variable) was associated with increased risk of BCR (7 studies, HR 1.04 (CI 1.02–1.05), I^2^ = 8%, *p* < 0.05) [3, 16–21] (Fig. 2). PSM length greater than 3 mm conferred a higher risk of BCR compared to less than 3 mm (4 studies, HR 1.99 (1.54–2.58) I^2^ = 0%, *p* < 0.05) (Fig. 3A) [14, 15, 22, 23]. PSM length greater than 3 mm increased the risk of BCR compared to negative surgical margin (6 studies, HR 2.25 (1.87–2.71) I^2^ = 24%, *p* < 0.001) [15, 23–27] (Fig. 3B). PSM length less than 3 mm also had higher risk of BCR compared to NSM (4 studies, HR 1.39 (1.03–1.87) I^2^ = 13%, *p* = 0.03) [15, 23–25] (Fig. 3C).

**Fig. 4:**
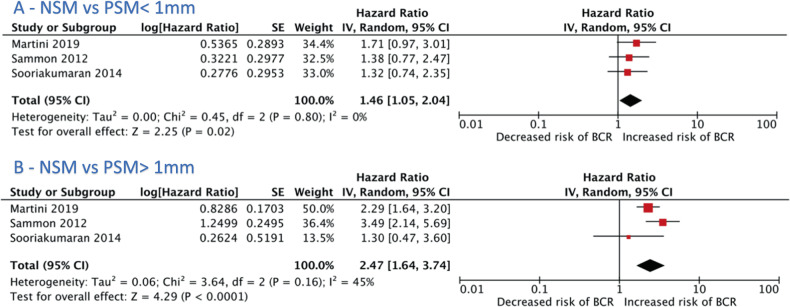
Biochemical recurrence risk for studies dichotomising margin length using a 1 mm threshold. **A** Compares BCR risk for studies comparing NSM and PSM length < 1 mm. **B** Compares BCR risk for studies comparing NSM and PSM > 1 mm. CI confidence interval, PSM > 1 mm positive surgical margin length greater than 1 mm, PSM < 1 mm positive surgical margin length less than 1 mm, NSM negative surgical margin.

There was only one study available for a reliable comparison between < 1 mm and > 1 mm length of PSM [29]; hence, a meta-analysis could not be performed. There was an increased risk of BCR associated with PSM length of less than 1 mm compared to NSM (3 studies, HR 1.46 (1.05–2.04), I^2^ = 0%, *P* = 0.02) [24, 26, 28] (Fig. 4A). PSM length greater than 1 mm also had a higher risk of BCR compared to NSM (3 studies, 2.47 (1.64–3.74) I^2^ = 45%, *p* < 0.001) [24, 26, 28] (Fig. 4B).

### Oncological outcomes

There was insufficient data in the identified studies to perform a meta-analysis for other oncological outcomes such as systemic progression and cancer-related mortality. One study found that PSM > 3 mm was associated with risk of clinical recurrence on imaging compared to NSM after multivariable analysis [24].

### Excluded studies

Several studies dichotomised patient groups using 1 mm PSM length but had several methodological differences preventing them from being included in the subgroup meta-analysis. Chapin et al. compared PSM lengths less than 1 mm, greater than 1 mm and NSM in men with organ-confined disease, where they noted that PSM length >1 mm increased the risk of BCR independently [30]. Shikanov et al. used a non-standard definition of BCR (> 0.1 ng/l) and compared NSM with margin <1 mm and >1 mm. They identified PSM <1 mm still had a profound impact on BCR. Given the nonstandard BCR definition, it is likely that there was an over-detection of BCR hence this was excluded from our analysis.

### Assessment of bias

**Fig. 5:**
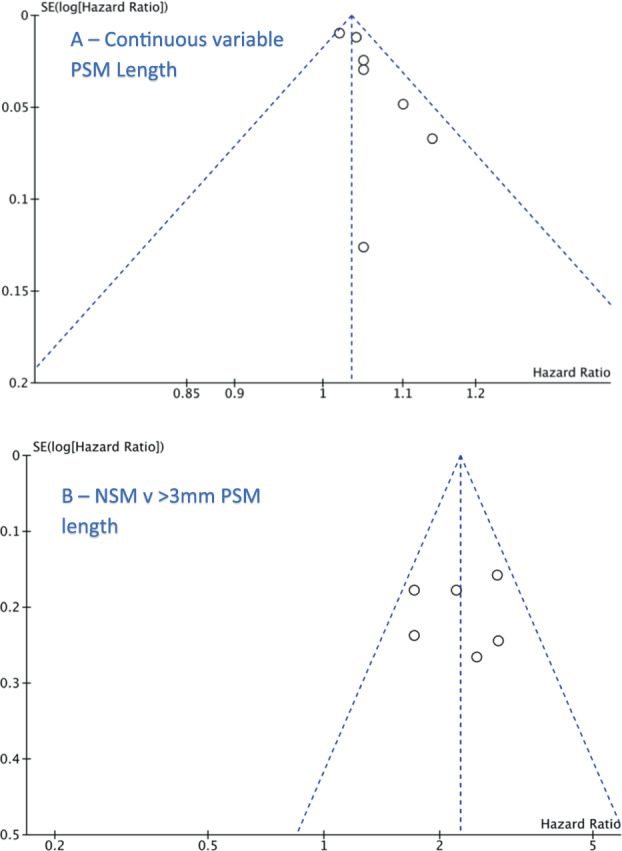
Funnel plot exploring publication bias. PSM positive surgical margin, NSM negative surgical margin, PSM > 3 mm positive surgical margin length greater than 3 mm.

All studies were non-randomized and retrospective in nature. Hence, there is a potential for selection bias given that adequate blinding of the outcomes to histology reviewers may not have been achieved. Most studies were classified as good quality based on the Newcastle Ottawa Scale, scoring lower if having inadequate follow-up time (<5 years). Most studies included in the meta-analysis adjusted for Gleason score, preoperative PSA and stage during multivariable analysis. Funnel plot analysis suggested minimal publication bias however the test is insufficient to distinguish chance from real asymmetry given that less than 10 studies were included (Fig. 5A, B).

## Discussion

PSM after radical prostatectomy is anecdotally considered an adverse outcome. However, only a subset of patients with PSM after radical prostatectomy experience adverse outcomes [5]. Further methods to classify, predict and assess the risk of progression in these patients are warranted so that secondary treatment can be initiated appropriately. In this meta-analysis, we demonstrate that an increase in PSM length is independently associated with an increased risk of BCR in men who have undergone radical prostatectomy. These results could improve the risk stratification of patients with a PSM. More specifically, higher PSM length (particularly PSM length ≥3 mm) may benefit from more frequent monitoring and consideration for adjuvant treatment. Shorter (<1 mm) PSM was associated with higher rates of BCR compared with negative margins, albeit with a lower risk than longer PSM; thus, we propose that these patients should be managed with surveillance and salvage radiation therapy if required, rather than adjuvant therapy as per recommendations from most guidelines [31]. Hence, this can help improve the oncological care for high-risk patients and spare unnecessary adverse effects of adjuvant therapy for lower-risk patients. Improved targeting of patients for adjuvant therapy could also provide economic advantages to the healthcare system by rationalising the significant costs associated with it, as noted by Martini et al. [32]. Further prospective and randomised controlled trials are still required to justify adjuvant therapy at a certain threshold. However, 3 mm could be used as a potential cut-off and basis for future trials. Based on our findings, we encourage institutions to report the length of the PSM and to consider this when counselling and determining surveillance or secondary treatments for these patients.

To our knowledge, this is the first systematic review to explore the length of PSM and its effect on BCR. There are several other systematic reviews published in regards to various features of PSM after radical prostatectomy. We have previously published another meta-analysis exploring the primary Gleason grade and Gleason grade group of the PSM [33]. This demonstrated that Gleason grade > 3 at the PSM and increasing Gleason grade group of PSM is independently associated with a higher risk of BCR. Yossepowitch et. al performed a systematic review in 2014 exploring outcomes associated with PSM after radical prostatectomy [34] and alluded to studies that demonstrated the prognostic implications of PSM length; however, a meta-analysis was not performed. A narrative review was also performed by Fontenot and Mansour mainly as a method to standardise reporting styles of pathological parameters of positive surgical margin [35]. Our review improves upon these earlier studies by objectively exploring the current evidence related to the impact of PSM length on BCR using a meta-analysis.

Since the studies included in this analysis assessed the impact of PSM length using various dichotomised groups, subgroup analyses were undertaken. For each subgroup, the difference in sample size and follow-up period were considered when determining the reliability of the results. It is important to note that most studies used the length of PSM as a continuous variable or dichotomised the PSM length using a 3 mm threshold. Hence, this subgroup analysis would be more reliable than those dichotomised using a 1 mm threshold. The use of 3 mm as a threshold was initially adopted by Babaian and colleagues based on the median length of PSM in their study cohort [36]. Brimo et al. also adopted it for similar reasons and noted that > 3 mm PSM length increased the risk of BCR compared to < 3 mm PSM length in a multivariable analysis; however, this study did not report a hazard ratio and hence was excluded from our analysis. Other reasons for exclusions include using a cohort only involving organ-confined disease and non-standard BCR definition [12, 30]. Several studies reported the length of PSM and BCR but failed to undertake multivariable analysis or report multivariable hazard ratio and hence were not included in the meta-analysis [37–42]. We specified a priori that preoperative PSA and pathological Gleason score are potential confounders and needed adjustment prior to inclusion in our meta-analysis. Several studies were identified during the search which could not be grouped into the corresponding subgroup analysis. Marcq et al. identified that men with apical PSM length greater than 3 mm had an increased risk of BCR compared to NSM after adjustment for stage, Gleason score and LN invasion, while apical PSM less than 3 mm did not identify any increased risk after multivariable adjustment over a median follow up of 7.6 years [43]. This study was not included in our review since it only included men with positive apical margins. Van Oort et al. dichotomised their results using a 10 mm PSM length, given their median PSM length of the cohort was 11 mm, and noted that PSM length >10 mm increased the risk of BCR compared to <10 mm [44]. Kir et al. performed a ROC analysis to determine the cut-off threshold. They showed that 2–3 mm, 3–6 mm, and >6 mm had a corresponding increased risk of BCR compared to NSM. 0–2 mm had equivalent risk compared to NSM hence concluded that a margin threshold of 2 mm should be used to identify a low and high-risk group [45]. Porpiglia et al. used a 2.8 mm threshold based on ROC analysis and identified that PSM length greater than 2.8 mm had an increased risk of BCR [46]. Saether et al. used a 6 mm threshold and showed that PSM length greater than 6 mm increased the risk of BCR but only on univariate analysis. This highlights significant disagreement between studies regarding the current margin length threshold for identifying a high and low-risk group. There are also some discrepancies in PSM length definitions among the studies. Some studies advocate grouping extensive positive surgical margin (Defined as >3 mm PSM length) with multifocal margins regardless of cumulative length and hence were excluded from the analysis [47, 48]. Stephenson et al. also noted extensive PSM associated with increased risk of BCR however used a non-objective definition of the presence of tumour at the margin in 1 section or more [49]. Overall, despite not being included in our meta-analysis, most of these studies support the use of margin extension as an independent prognostic factor to identify the risk of BCR in men with positive surgical margins.

The review’s strengths include that it followed a protocol published before the literature searches commenced, the use of data extracted from studies reporting a multivariable analysis only, minimal heterogeneity between the studies for various subgroup analyses and the incorporation of various dichotomised thresholds of PSM length. Limitations include a low number of studies for certain subgroup meta-analyses, certain studies included with follow-up of less than 5 years and a lack of randomised controlled trials, meaning that the studies included may be prone to selection biases. This risk was assessed using the Newcastle Ottawa Scale, with most studies receiving a score of 3 for the selection component (most points were lost for insufficient blinding and potential selection bias due to retrospective reviews) (Table 2). It is also important to note that the results may potentially be influenced by reporting bias. Emerson 2005 identified PSM length as a risk factor but failed to demonstrate it in multivariable analysis and did not report the multivariable hazard ratios and was therefore excluded from our meta-analysis [42]. The heterogeneity (I^2^) among most of the analyses was minimal (under 15%), with one subgroup analysis being 45% (Fig. 4B). This could be accounted for by differences in the follow-up period and technique (robotic, perineal, laparoscopic, or open) between these cohort studies. Only a few studies adjusted for other pathological parameters such as Gleason score or grade of the PSM and lymph node status. These are also important parameters that need to be considered when evaluating BCR risks as suggested by some studies, including our previous meta-analysis [14, 33, 50]. Future studies should consider these variables and also the location of the PSM [35]. These factors need to be considered when modelling a high and low-risk group in this population of men. For example, a Gleason grade 3 and PSM length of 4 mm margin would be deemed more favourable compared to a Gleason 4/5 and PSM length of 3 mm. Another weakness is the use of BCR as the clinical endpoint; clinical progression by imaging would be a more relevant and reliable endpoint since it is much more closely associated with cancer-related mortality. Only one study identified an increased risk of clinical progression (on imaging) with PSM length greater than 3 mm [24]. This study did not identify any significant risk of cancer progression for PSM length less than 3 mm compared to negative surgical margins however it was limited by a three-year follow-up period. Hence, we expect further long-term studies exploring BCR and other oncological outcomes, particularly systemic progression, cancer-specific mortality, and overall survival.

**Table 2:** Newcastle-Ottawa Quality assessment for included studies.

Study	Selection	Comparability	Outcome	Interpretation based on AHRQ standards (Good/Fair/Poor)
Cao et al. [16]	***	*	**	Good
Holleman et al. 2019	***	*	**	Good
Hsu et al. [18]	***	*	***	Good
Huang et al. [19]	***	*	**	Good
Kates et al. [20]	***	*	**	Good
Kim et al. [21]	***	*	**	Good
Savdie et al. 2011	***	*	***	Good
Dev et al. [15]	***	*	**	Good
Ploussard et al. [22]	***	*	**	Good
Preisser et al. [14]	***	*	***	Good
Sevoll et al. 2014	***	*	***	Good
Lee et al. [25]	***	*	**	Good
Maxeiner et al. [27]	***	*	***	Good
Martini et al. 2019	***	*	**	Good
Sammon et al. 2012	***	*	**	Good
Sooriakumaran et al. 2014	***	*	***	Good

## Conclusion

Positive surgical margin length is independently prognostic for biochemical recurrence in patients after radical prostatectomy. Further long-term studies are needed to estimate the impact of these variables on cancer-specific outcomes such as systemic progression and mortality and if a low-risk margin threshold can be safely established.

## Data Availability

The datasets generated during and/or analysed during the current study are available from the corresponding author on reasonable request.
